# Biotransformation of Monocyclic Phenolic Compounds by *Bacillus licheniformis* TAB7

**DOI:** 10.3390/microorganisms8010026

**Published:** 2019-12-21

**Authors:** Enock Mpofu, Joydeep Chakraborty, Chiho Suzuki-Minakuchi, Kazunori Okada, Toshiaki Kimura, Hideaki Nojiri

**Affiliations:** 1 Biotechnology Research Center, The University of Tokyo, 1-1-1 Yayoi, Bunkyo-ku, Tokyo 113-8657, Japan; 2 Collaborative Research Institute for Innovative Microbiology, The University of Tokyo, 1-1-1 Yayoi, Bunkyo-ku, Tokyo 113-8657, Japan; 3 Agriculture and Biotechnology Business Division, Toyota Motor Corporation, 1099 Marune, Kurozasa-cho, Miyoshi-shi, Aichi 470-0201, Japan

**Keywords:** agro-industrial wastes, allelochemicals, *Bacillus licheniformis*, biotransformation, phenolic compounds

## Abstract

*Bacillus licheniformis* strain TAB7 is a bacterium used as a commercial deodorizing agent for compost in Japan. In this work, its ability to biotransform the following monocyclic phenolic compounds was assessed: ferulate, vanillate, *p*-coumarate, caffeate, protocatechuate, syringate, vanillin, and cinnamate (a precursor for some phenolic compounds). These compounds are abundant in composting material and are reported to have allelopathic properties. They come from sources such as plant material decomposition or agro-industrial waste. Biotransformation assays were carried out in LB supplemented with 0.2 mg/mL of an individual phenolic compound and incubated for up to 15 days followed by extraction and HPLC analysis. The results showed that TAB7 could biotransform ferulate, caffeate, *p*-coumarate, vanillate, protocatechuate, and vanillin. It, however, had a poor ability to transform cinnamate and syringate. LC-MS/MS analysis showed that ferulate was transformed into 4-vinylguaiacol as the final product, while caffeate was transformed into 4-ethylcatechol. TAB7 genome analysis suggested that, while TAB7 may not mineralize phenolic compounds, it harbored genes possibly encoding phenolic acid decarboxylase, vanillate decarboxylase, and some protocatechuate degradation pathway enzymes, which are involved in the catabolism of phenolic compounds known to have negative allelopathy on some plants. The results thus suggested that TAB7 can reduce such phenolic compounds in compost.

## 1. Introduction

*Bacillus licheniformis* strain TAB7 is a thermophile that has been isolated as a Tween 20 degrader from composting livestock excrement in Japan [1]. It was subsequently shown to have the ability to deodorize short-chain fatty acids such as valeric, butyric, propionic, and related acids responsible for the offensive odor in manure. Toyota Motor Corporation and Menicon Co., Ltd. jointly developed a composting process called “resQ45” in which TAB7 is used as a compost-deodorizing agent [2].

The use of TAB7 in composting, potentially leading to unusually high TAB7 load in compost and environments where TAB7-deodorized compost has been introduced, has raised some important questions on what other effect(s) the bacteria could potentially have on compost and the environment. It is in this context that the ability of TAB7 to biotransform phenolic compounds was assessed. The ability of TAB7 to biotransform phenolic compounds such as monocyclic hydroxycinnamates (e.g., ferulate and caffeate) and hydroxybenzoates (e.g., vannilate, protocatechuate and vanillin) could potentially have important implications because these phenolic compounds are important environmental pollutants [3] abundant in composting material and whose role in negative allelopathy has long been established [4,5,6,7,8,9]. Their origin is both natural and industrial. Plants naturally produce phenolic compounds as monomers of lignin, tannins, and other polyphenols, and these can be released into the environment as products of plant decay or root exudates [3,6]. Plant-based industries such as wood debarking, cork making, wineries, tea, coffee production, kraft pulp making, and related industries also release phenolic compounds as waste into the environment [3]. Monocyclic phenolic compounds such as ferulate, *p*-coumarate, caffeate, syringate, vanillate, and others are known to be among the most important and abundant class of allelochemicals [4]. Previous research has established that the phytotoxicity of phenolic compounds is dependent on their chemical and/or physical properties and the presence of other toxic or non-toxic biomolecules [7,8]. Some phytotoxic phenolic compounds show synergy in their phytotoxicity lowering their inhibitory threshold [7,8].

The fate of phenolic compounds in the environment is determined by both biotic and abiotic processes [9]. Biotic processes involve microorganisms biotransforming these allelochemicals into compounds with different biochemical properties, rendering them less or non-phytotoxic. Thus, the aim of this research was to investigate the ability of TAB7 to biotransform this class of abundant industrial and natural pollutants with allelochemical properties. The monocyclic phenolic compounds used in this study are ferulate, caffeate, *p*-coumarate, vanillate, syringate, protocatechuate, vanillin, and cinnamate, chosen primarily because of their reported contribution to negative allelopathy [4,5,6,7,8,9].

## 2. Materials and Methods

### 2.1. Bacterial Strain

*B. licheniformis* strain TAB7 has been isolated as a Tween 20-degrader from composting manure in Japan [1]. The whole-genome sequence of this organism has been determined [10]. *B. licheniformis* JCM 2505^T^, a type strain, was purchased from the Japan Collection of Microorganisms (RIKEN-BRC, Tsukuba, Japan).

### 2.2. Chemicals and Reagents

Ferulate, *p*-coumarate, caffeate, protocatechuate, syringate, cinnamate, vanillate, vanillin, ethyl acetate, and methanol (HPLC and LC-MS/MS grades) were purchased from Wako Pure Chemical Industries Ltd., (Tokyo, Japan). DMSO was bought from Kanto Chemical Co., Inc. (Tokyo, Japan). Yeast extract and agar were purchased from Nacalai Tesque Inc. (Kyoto, Japan). Tryptone was purchased from Becton, Dickinson and Company (Tokyo, Japan).

### 2.3. Utilization of Phenolic Compounds as Sole Carbon Sources

TAB7 cells from stock culture stored at −80 °C were streaked on LB-agar (g/L: 10 tryptone, 5 yeast extract, 10 NaCl, 16 agar) [11] and incubated overnight at 30 °C. A colony was picked and inoculated into 5 mL of LB broth (LB medium without agar) and incubated at 30 °C overnight with reciprocal shaking (300 strokes/min). Cells were harvested by centrifugation (5000× *g* for 5 min) using a sterile centrifuge tube and washed three times with equal volume (i.e., 5 mL) of a carbon-free (CF) buffer containing the following in g/L: 2.2 Na_2_HPO_4_, 0.8 KH_2_PO_4_, 3.0 NH_4_NO_3_. Cells were then re-suspended in 5 mL of CF buffer. One hundred microliters of this cell suspension were then inoculated into separate 5 mL portions of sterile CF-buffer containing micronutrients (in g/L: 0.01 FeCl_3_·6H_2_O, 0.2 MgSO_4_·7H_2_O, and 0.01 CaCl_2_·6H_2_O), with a final concentration of 0.5 mg/mL of one phenolic acid added from stock solution (12 mg/mL stock solutions prepared by dissolving respective phenolic compound in DMSO). These were then incubated at 30 °C in a Taitec OD monitor (Taitec Co. Ltd., Aichi, Japan) by shaking (200 strokes/min) for up to 48 h. For the control, 0.5 mg/mL of glucose was added instead of a phenolic compound.

### 2.4. Biotransformation Assays

TAB7 seed culture was prepared as described above, although, in this experiment, the seed culture was not washed. One hundred microliters of the overnight culture were directly inoculated into separate 10 mL portions of sterile LB broth. A final concentration of 0.2 mg/mL of one phenolic compound (prepared as described above) was added and incubated at 30 °C by shaking for up to 14 days (depending on the compound) taking 2 mL of samples at appropriate times for HPLC analysis. For negative control, phenolic compounds were added to TAB7-free, sterile media separately and incubated under the same condition.

### 2.5. Analytical Procedure

#### 2.5.1. Sample Preparation for HPLC Analysis

The same method was employed for all samples. Two mL of cell suspension (or culture) supplemented with each phenolic compound were centrifuged (5000× *g* for 5 min) to remove the cells, and a final concentration of 0.2 mg/mL of an internal standard (from stock solution dissolved in DMSO) was added to the supernatant. Protocatechuate was used as an internal standard for analysis of all other phenolic compounds. For protocatechuate analysis, vanillate was used as an internal standard. Resultant supernatants were then acidified with 2N HCl to approximately pH 2. Extraction was done three times with equal volumes (2 mL) of ethyl acetate. After extraction, ethyl acetate fractions were completely evaporated by Taitec Spin Dryer Standard VC-96R centrifugal evaporator (Taitec Co. Ltd.), and residues were dissolved in 2 mL of methanol, filtered through 0.22 µm (PTFE) filters (Merck Millipore) and subjected to HPLC analysis.

#### 2.5.2. HPLC Analysis

A Hitachi Elite LaChrom L2455 High-Performance Liquid Chromatography (HPLC) system (Hitachi, Ltd., Tokyo, Japan) equipped with a diode array detector (DAD), an autosampler injector, a thermostat column compartment, and a PEGASIL-B ODS (octadecyl silica) analytical column (4.6 mm × 250 mm) (Senshu Scientific Co., Ltd., Tokyo, Japan) was used. The mobile phase was a mixture of methanol (A) and water containing 2% (*v*/*v*) acetic acid (B). The following gradient elution profile was used: 15–40% A (0.0–22.5 min), 40% A (22.5–25.0), 40–15% A (25.0–30.0), 15% A (30.0–40.0). The flow rate was 1 mL/min, and the injection volume was 30 μL. Oven temperature during the analysis was maintained at 40 °C. The detection wavelength was set at 254 nm.

#### 2.5.3. LC-MS /MS Analysis of Ferulate and Caffeate Degradation Products

To identify the products formed from ferulate and caffeate, potential products were used as comparative standards in LC-MS/MS analysis. The following compounds were used as potential products (and hence as standards): ferulate, caffeate, *p*-coumarate, vanillate, protocatechuate, vanillin, catechol, guaiacol, 4-vinylguaiacol, and 4-ethylcatechol. A mini-library of masses of standards’ precursor and product ions was created by directly infusing 10 ppm (for guaiacol) or 0.2 ppm (for the rest of the compounds) of each standard (dissolved in methanol), one at a time into the ESI-MS/MS and acquiring its precursor (Q1) and product (Q3) ion masses. The method thus created was validated by running the same standards. After validation, phenolic compound degradation products were determined by running samples against the created mini-library of standards. The LC-MS/MS system used comprised an AB SCIEX API-3000 spectrometer (AB Sciex LLC., Redwood City, CA, USA) with an electrospray ion (ESI) source and an Agilent 1100 HPLC instrument (Agilent Technologies, Inc., Santa Clara, CA, USA) equipped with a Pegasil ODS SP100 C18 column (length 150 mm, diameter 2.1 mm) (Senshu Scientific Co., Ltd., Tokyo, Japan). The injection volume was 5.0 μL, and flow rate was kept at 0.2 mL/min. Analysis was done in negative ion mode. The mobile phase was the same as in HPLC, and the following gradient elution profile was used: 15–40% A (0.0–11.5 min), 40–100% A (11.5–13.0), 100% A (13.0–17.5), 100–15% A (17.5–22.5), 15% A (22.5–30.0).

## 3. Results and Discussion

### 3.1. Ability to Utilize Phenolic Compounds as Sole Carbon Sources

Firstly, we assessed the ability of TAB7 to utilize the following phenolic compounds as sole carbon sources; ferulate, caffeate, *p*-coumarate, vanillate, protocatechuate, syringate, and vanillin. After incubation in carbon-free medium supplemented with each phenolic compound (separately) at 30 °C for 48 h, there was no increase in OD_600_ for all phenolic compounds, yet there was growth in the control (glucose) (data not shown). These results suggested that TAB7 could not utilize these phenolic compounds as sole carbon sources.

### 3.2. Transformation Ability for Phenolic Compounds

**Figure 1:**
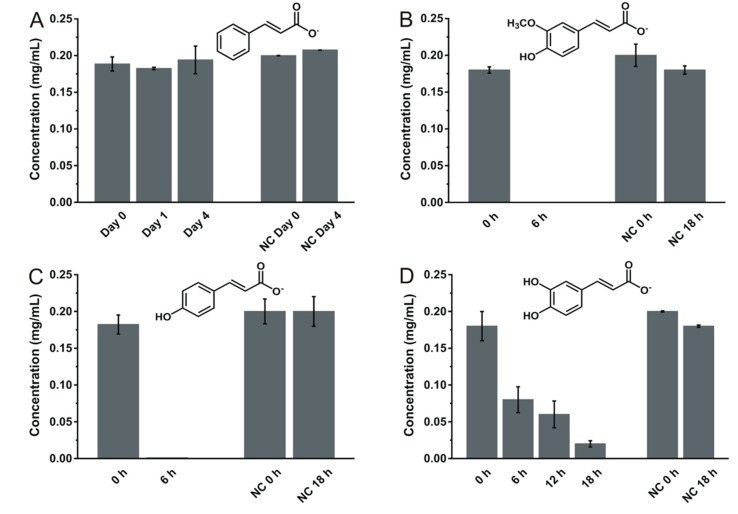
Biotransformation of cinnamate (**A**) and its hydroxyl derivatives, ferulate (**B**), *p*-coumarate (**C**), and caffeate (**D**) by the *B. licheniformis* strain TAB7. Data are expressed as means ± standard deviation from triplicates. NC: negative control. Relevant chromatograms are shown in Figure S1, found in the Supplementary Data File.

The biotransformation ability of TAB7 was assessed for seven phenolic compounds: viz. ferulate, caffeate, *p*-coumarate (hydroxycinnamates), vanillate, protocatechuate, syringate, and vanillin (hydroxybenzoates) and one non-phenolic compound, cinnamate, which is an important precursor of hydroxycinnamates. TAB7 could transform ferulate, *p*-coumarate (within 6 h), and caffeate (within 24 h) as shown in Figure 1. It, however, could not transform cinnamate, their precursor compound in four days of incubation, suggesting the possibility that TAB7 enzyme(s) involved in these biotransformation steps needed at least a hydroxyl group at the *para* or *meta* position of the benzyl moiety of the compound in order to metabolize it.

**Figure 2:**
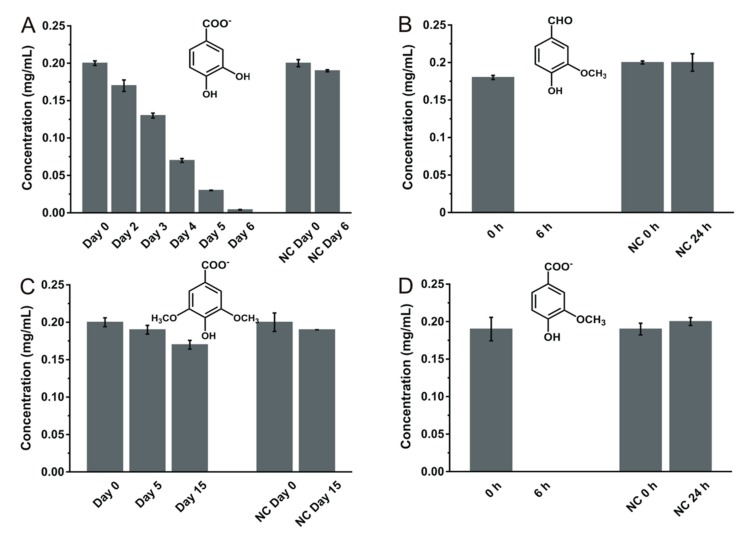
Biotransformation of hydroxybenzoates such as protocatechuate (**A**), vanillin (**B**), syringate (**C**), and vanillate (**D**) by the *B. licheniformis* strain TAB7. Data are expressed as means ± standard deviation from triplicates. NC: negative control. Relevant chromatograms are shown in Figure S1, found in the Supplementary Data File.

On the hydroxybenzoates front, TAB7 could transform vanillate and vanillin within 6 h and protocatechuate in 7 days of incubation, while there was no significant decrement in syringate even after 15 days of incubation (Figure 2). The poor ability of TAB7 to transform syringate could perhaps be attributed to the effect of steric hindrance by more than two functional groups on the benzene ring. In order to determine if this transformation ability pattern was unique to TAB7 in *B. lichenifomis* species, we assessed the ability of *B. licheniformis* JCM 2505^T^, a type strain, to transform the same phenolic compounds. The results showed a markedly similar degradation pattern (Figure S2, Supplementary Data File). Type strain JCM 2505^T^ transformed all phenolic compounds that were transformed by TAB7, indicating that TAB7 is not unique in its ability to transform the compounds employed in this study.

In the biotransformation of ferulate, caffeate, and *p*-coumarate, we detected new peaks, which appeared with a concomitant decrease or disappearance of substrate peaks. These peaks represented either the intermediates or products that were formed during biotransformation experiments of these phenolic compounds.

### 3.3. Identification of Intermediates/Products by LC-MS/MS

To identify these intermediates/products, we conducted LC-MS/MS analysis. Firstly, we validated the method we created by running standards (10 ppm for guaiacol and 0.2 ppm for the rest of the standards) one by one and combined in LC-MS/MS. The results showed that the method could reliably detect the standards at such low concentrations (Figure S3, Supplementary Data File). Guaiacol, however, was undetectable at 0.2 ppm.

#### 3.3.1. Identification of the Ferulate Biotransformation Product

**Figure 3:**
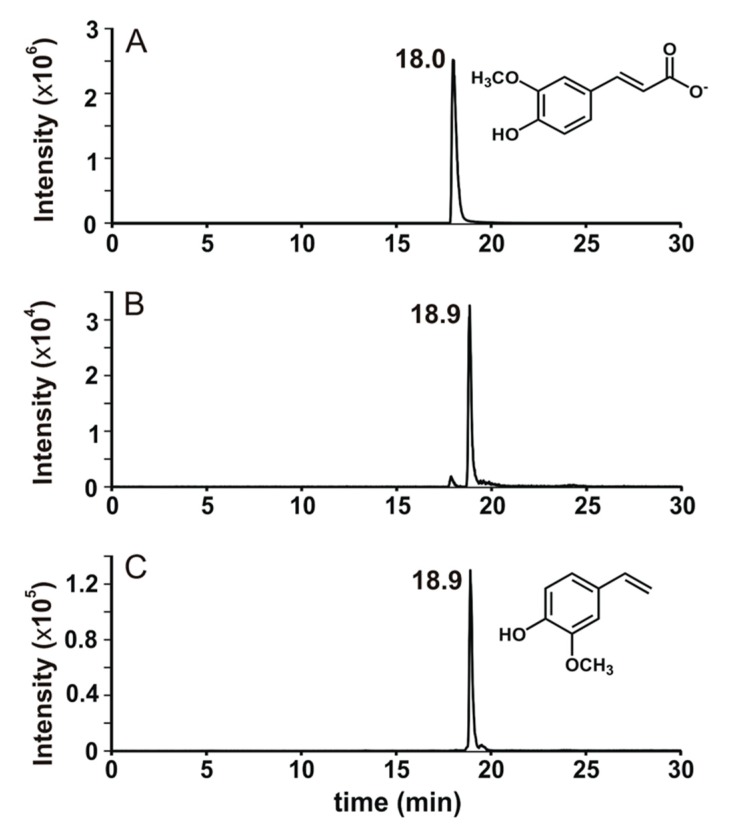
LC-MS/MS analysis of standard ferulate (**A**) and biotransformation product of ferulate by strain TAB7 (**B**), which is identical to that of standard 4-vinylguaiacol (**C**).

In ferulate biotransformation analysis, there was only one new peak, which appeared earlier than 6 h in LB broth. The peak height and area remained the same for up to 2 days, suggesting that the compound responsible for this peak was not an intermediate in ferulate biotransformation but a final product. LC-MS/MS analysis of this peak showed that it was 4-vinylguaiacol (Figure 3).

Several microorganisms have been reported to decarboxylate ferulate to 4-vinylguaiacol, and some can even further transform 4-vinylguaiacol to 4-ethylguaiacol [12,13,14,15,16]. While most of the reported organisms convert ferulate to other compounds like vanillin, vanillate, and protocatechuate via either 4-vinylguaiacol [17] or feruloyl-coA [18], TAB7 seems to convert ferulate to 4-vinylguaiacol as the final product. This conversion is catalyzed by phenolic acid decarboxylase (PAD), an enzyme known to decarboxylate ferulate, *p*-coumarate, and caffeate to their vinyl derivatives [19]. This, however, is not unique to TAB7 [14]. TAB7 conversion of ferulate to 4-vinylguaiacol could be important because 4-vinylguaiacol is less implicated in seed germination and plant growth inhibition [20,21,22]; thus, TAB7 may not only deodorize compost but can also improve it by reducing allelochemicals in compost cooperatively with other bacteria. The biotransformation profile (in HPLC) of *p*-coumarate biotransformation was markedly similar to that of ferulate (see Figure S1, Supplementary Data File). Hence, it seemed reasonable to infer that *p*-coumarate was decarboxylated to 4-vinylphenol.

#### 3.3.2. Identification of Caffeate Biotransformation Product

**Figure 4:**
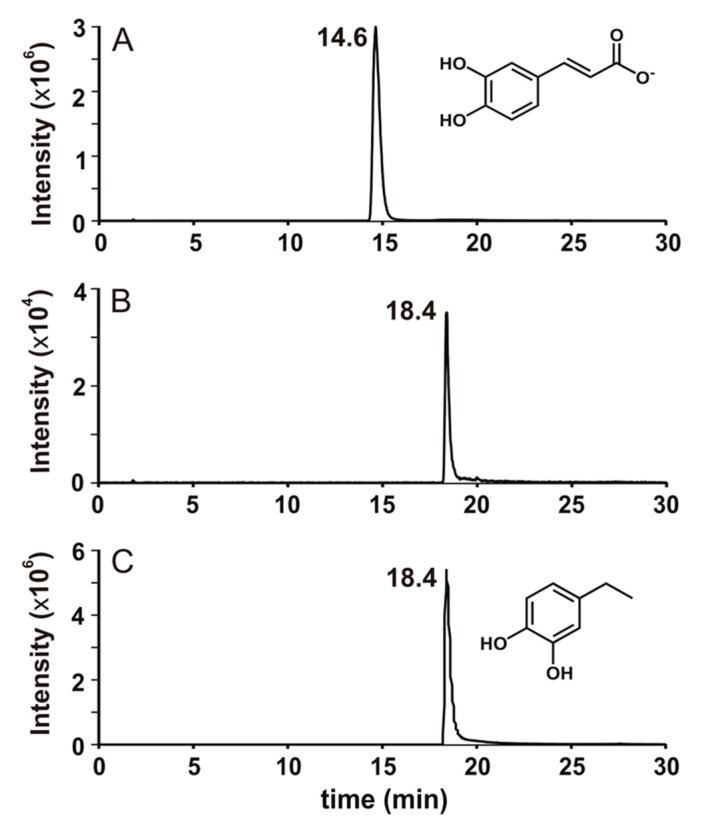
LC-MS/MS analysis of standard caffeate (**A**) and biotransformation product of caffeate by strain TAB7 (**B**), which is identical to that of standard 4-ethylcatechol (**C**).

**Figure 5:**
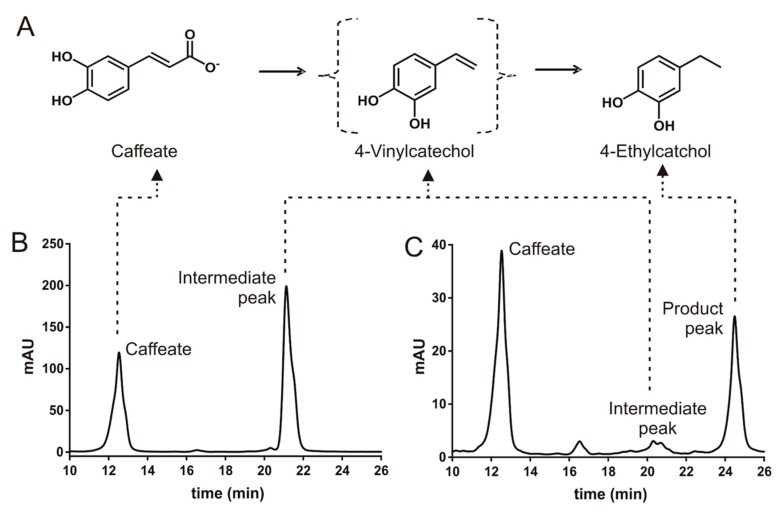
Putative biotransformation pathway of caffeate by *B. licheniformis* TAB7 (**A**), identified on the basis of HPLC analysis of culture extracts obtained after incubation with caffeate for 12 h (**B**) and 18 h (**C**) respectively. 4-Ethylcatechol was detected and identified by LC-MS/MS.

Although the only difference between caffeate and ferulate is the methylation of 4-hydroxyl group on the catechol moiety, the biotransformation of caffeate by TAB7 showed a different and interesting production pattern of intermediate/product (Figure 4; Figure 5). In the HPLC analysis of caffeate biotransformation by TAB7, two new peaks appeared consecutively. One appeared at the retention time (Rt) around 21 min after 6 h of incubation, which remained unchanged until after 12 h of incubation but disappeared after 18 h of incubation. Another peak with an Rt of around 24 min then appeared (Figure 5). This suggested that the first peak could be an intermediate, while the second one could either be a product peak or another intermediate that may be produced from the intermediate observed at the Rt of around 21 min.

According to reports by Senger et al. [23], Buron et al. [24], and Peppercorn and Goldman [25], the first peak at the Rt of 21 min was thought to possibly be 4-vinylcatechol and the second peak at the Rt of 24 min was putatively thought to be 4-ethylcatechol. While we could not get a commercial standard for 4-vinylcatechol, 4-ethylcatechol was commercially available. We, therefore, carried out LC-MS/MS analysis of the samples extracted from the 18 h biotransformation assay of caffeate to identify the product. The results confirmed 4-ethylcatechol as the compound responsible for the second peak (Figure 4 and Figure 5). 4-Ethylcatechol could be the final product, or it could be converted into another compound not detectable in the HPLC or the LC-MS/MS methods used in this study.

Buron et al. [24] and Peppercorn and Goldman [25] suggested that caffeate can be transformed to 4-ethylcatechol via 4-vinylcatechol by some human gut bacteria. This seems to be the same conversion carried out by TAB7 (Figure 5). In this putative degradation pathway, caffeate is firstly converted to 4-vinylcatechol via the action of a putative phenolic acid decarboxylase. 4-Vinylcatechol is then converted to 4-ethylcatechol by putative 4-vinylcatechol reductase. Until recently (June 2018), when Santamaria et al. described ethylphenol formation by *Lactobacillus plantarum* [26], there had been no report on the characterization of bacterial enzyme responsible for reducing vinyl derivatives of phenolic compounds into their corresponding ethyl forms, while there has been considerable research on fungal vinyl derivative reductases, principally from *Brettanomyces bruxellensis* [27,28,29]. While the vinyl derivative reductases described so far seem to convert all vinylguaiacol, vinylcatechol, and vinylphenol into their corresponding ethyl derivatives, it seems that the putative enzyme in TAB7 has more specificity for vinylcatechol, or, unlike others, it may be specifically induced by either caffeate or its products. Thus, the putative degradation pathway of caffeate by TAB7 could add further basic knowledge of bacterial conversion of vinyl derivatives of phenolic compounds to their corresponding ethyl forms.

Caffeate, *p*-coumarate, and ferulate are reported to be decarboxylated by phenolic acid decarboxylase (PAD) into their vinyl-derivatives (for conversion scheme summary, see Figure S4, Supplementary Data File) [19]. We did not carry out LC-MS/MS analysis of the *p*-coumarate transformation; however, it seemed reasonable to infer from caffeate and ferulate transformation how *p*-coumarate could be transformed. It is likely that TAB7 decarboxylates *p*-coumarate to 4-vinylphenol, which may further be converted to its ethyl derivative.

### 3.4. Phenolic Compounds Degradative Genes in TAB7

**Figure 6:**
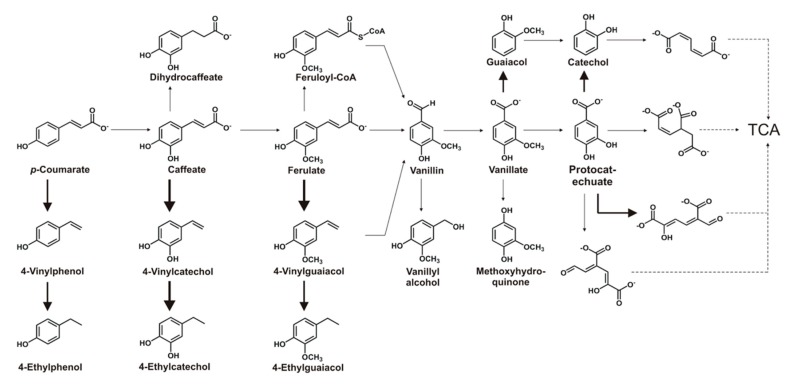
The network of pathways involved in degradation of phenolic compounds showing protocatechuate as central intermediate towards the TCA cycle. The thickest arrows represent biotransformation steps in TAB7 confirmed by the presence of putative genes and product detection when TAB7 was grown in LB media supplemented with the respective substrate. Intermediate arrows indicate biotransformation steps inferred due to the presence of putative genes and the disappearance of the respective substrate. The thinnest arrows indicate steps whose putative genes are not yet identified in TAB7. Dashed arrows indicate transformations involving more than one step.

In the degradation of most phenolic compounds, protocatechuate is a central intermediate as the degradation moves towards the tricarboxylic acid (TCA) cycle, as shown in Figure 6 [30,31]. Using the wealth of knowledge on bacterial degradation of aromatic compounds [30,31,32,33,34,35], characterized genes in protocatechuate degradation pathways were used to search and analyze the TAB7 genome for protocatechuate degradative genes, gene clusters, or operons. It was found that TAB7 seems to have an incomplete protocatechuate *para*-cleavage pathway (Figure S5, Supplementary Data File).

In this pathway, protocatechuate is cleaved to 5-carboxy-2-hydroxymuconate-6-semialdehyde by protocatechuate 2,3-dioxygenase and proceeds towards the TCA cycle. The TAB7 genome has all putative genes in this pathway (*praABCDEHI*) (Figures S5 and S6 and Table S1, Supplementary Data File) except for the last two leading into the TCA cycle, i.e., *praF* (coding for 4-hydroxy-2-oxovalerate aldolase) and *praG* (coding for acetaldehyde dehydrogenase). This means that TAB7 could mineralize protocatechuate either by *para*-cleavage pathway with some modification(s) or other pathway(s). In the former case, TAB7 may have alternative enzymes capable of channeling the pathway into the TCA cycle or other metabolic pathways. These could be enzymes involved in other pathways but have broader substrate specificity to also promiscuously participate in the protocatechuate degradation pathway. The incomplete protocatechuate degradation pathway seems to validate experimental results on the utilization of phenolic compounds as sole carbon sources (Figure S2, Supplementary Data File). It is thus expected that TAB7 does not have the ability to utilize protocatechuate or other phenolic compounds whose degradation pathways are linked to the protocatechuate degradation pathway as sole carbon sources.

In addition to the protocatechuate *para* cleavage pathway genes, TAB7 also has a gene encoding a putative vanillate decarboxylase, which shows 62.5% identity with the query (GenBank AAD28782). This enzyme may decarboxylate vanillate to guaiacol. Furthermore, TAB7 has a putative aldehyde dehydrogenase, which shows 66.1% identity to a *Geobacillus stearothermophilus* aldehyde dehydrogenase (GenBank BAA02975), whose product may catalyze the conversion of vanillin to vanillate. However, a putative *O-*demethylase, which catalyzes the removal of a methyl group from a methoxy group, has not been found in the TAB7 genome. This would suggest that TAB7 might not have an ability to demethylate ferulate, vanillate, and guaiacol to caffeate, protocatechuate, and catechol, respectively. This could further suggest that ferulate and vanillate, as well as guaiacol, cannot be channeled into the TCA cycle via the known protocatechuate degradation pathways. Thus, it is likely that TAB7 does not mineralize these phenolic compounds, at least not using the hitherto known pathways.

TAB7 also harbors a putative copper–zinc superoxide dismutase (Cu–Zn–SOD), which shares the same conserved domains with the Cu–Zn–SOD/vinylphenol reductase in *Brettanomyces bruxellensis* (Figure S7, Supplementary Data File), although the percentage identity with the query (GenBank: KX752264) is low (30% identity and 72% query cover), and the same conserved domains suggest a similar function. This putative vinylphenol reductase in *Brettanomyces bruxellensis* has both superoxide dismutase and vinylphenol reductase activities [36]. Thus, it may be conceivable that the putative 4-vinylcatechol reductase in TAB7 responsible for producing 4-ethylcatechol in caffeate degradation is in fact a Cu–Zn–SOD. Searching homologous genes for other phenolic compounds degradation pathways, such as the gallate degradation pathway (*galABCDPRT*) [37] and the syringate degradation pathway [38], suggested that TAB7 lacks putative enzymes for these pathways. From the genome analysis of TAB7, it appears that TAB7 can biotransform some allelopathic phenolic compounds such as ferulate, *p*-coumarate, caffeate, vanillate, protocatechuate, and vanillin into other organic compounds possibly with less or no negative allelopathy.

## 4. Conclusions

In this paper, we show, through experimental work, that the *Bacillus licheniformis* strain TAB7, which is used as a deodorizing agent for compost, has the ability to biotransform some monocyclic phenolic compounds found in abundance in compost and are widely implicated in negative allelophathy. It, however, cannot utilize them as sole carbon sources. TAB7 genome analysis corroborates experimental work by showing that the organism has putative genes whose products are involved in catabolism of phenolic compounds, but that it has an incomplete protocatechuate degradation pathway, which may be why it cannot utilize phenolic compounds as sole carbon sources. Since all these monocyclic phenolic compounds have been implicated in allelopathy, TAB7 may, in this way, improve the quality of compost by reducing plant growth inhibitors in the compost cooperatively with other bacteria.

## Supplementary Materials

The following are available online at https://www.mdpi.com/2076-2607/8/1/26/s1. Figure S1: HPLC chromatograms of culture extracts of strain TAB7 incubated individually with different phenolics. Figure S2: Biotransformation of hydroxycinnamates and hydroxybenzoates by *B. licheniformis* JCM 2505 type strain. Figure S3: Standards run in LC-MS/MS using the method created for analysis of ferulate and caffeate biotransformation intermediate/products by strain TAB7. Figure S4: Conversion scheme of ferulate, *p*-coumarate and caffeate into their vinyl and ethyl-derivatives. Figure S5: Protocatechuate degradation pathway found TAB7. Figure S6: Gene organization of the *pra* gene cluster in *B. licheniformis* TAB7 and *Paenibacillus* sp. JJ-1b. Figure S7: Comparison of conserved domains present in venylphenol reductase/superoxide dismutase from *Brettanomyces bruxellensis* (GenBank: KX752264) and putative venylcatechol reductase/superoxide dismutase from strain TAB7 chromosome (Locus tag AYC51877). Table S1: Putative genes involved in degradation of protocatechuate by strain TAB7.
