# Supplementary material for: Biotransformation of Monocyclic Phenolic Compounds by Bacillus licheniformis TAB7
Source: Microorganisms. 2019 Dec 21;8(1):26. doi: 10.3390/microorganisms8010026 (PMC7022639; doi:10.3390/microorganisms8010026)
Supplement: Supplementary file 1 [file microorganisms-08-00026-s001.pdf]

Supplementary Data File

Biotransformation of Monocyclic Phenolic Compounds by *Bacillus licheniformis* TAB7

Enock Mpofu [1,2], Joydeep Chakraborty [1], Chiho Suzuki-Minakuchi [1,3], Kazunori Okada [1], Toshiaki Kimura [4], and Hideaki Nojiri [1,3,*]

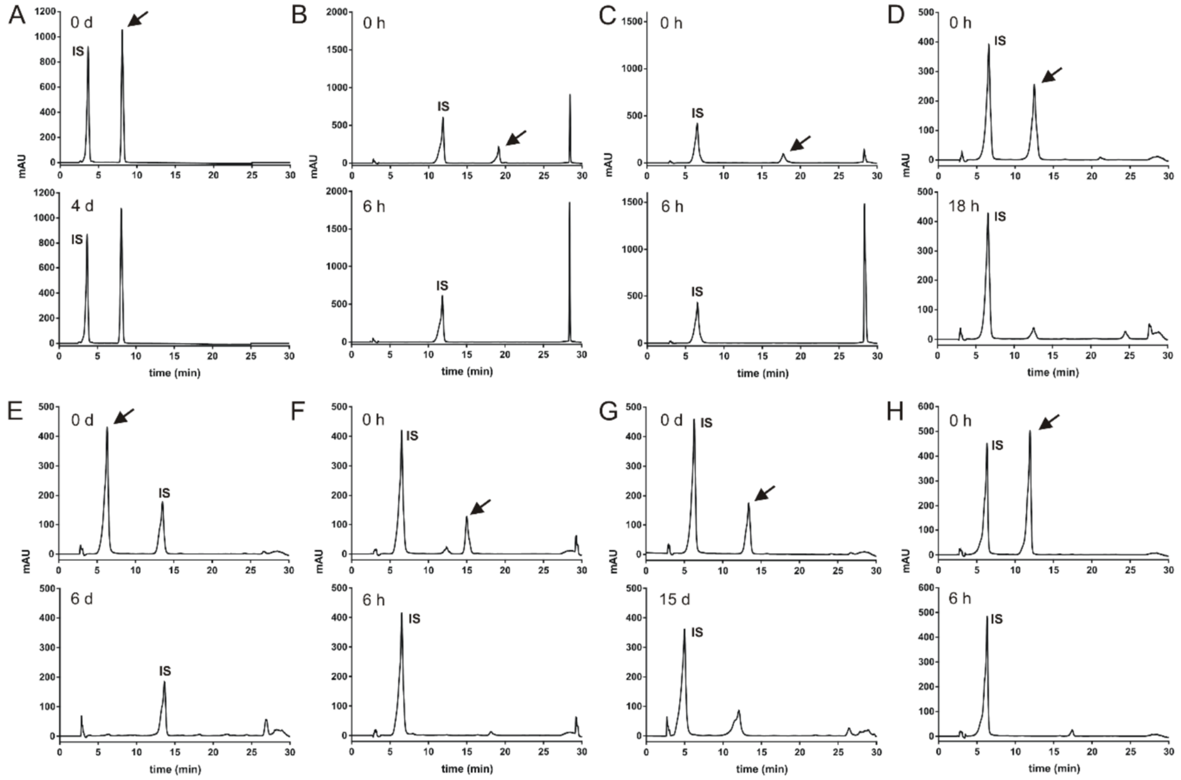

Figure 1. HPLC chromatograms of culture extracts obtained from incubation of *B. licheniformis* strain TAB7 with phenolic substrates, viz. cinnamate (A), ferulate (B), *p*-coumarate (C), caffeate (D), protocatechuate (E), vanillin (F), syringate (G) and vanillate (H). For each substrate, the upper chromatogram corresponds to the initial time point of incubation while the lower one corresponds to the final time point, as shown in Figures 1 and 2 in the main text. IS indicates the peak of the internal standard used for quantitation of substrate concentration, while the arrow indicates the peak of the substrate.

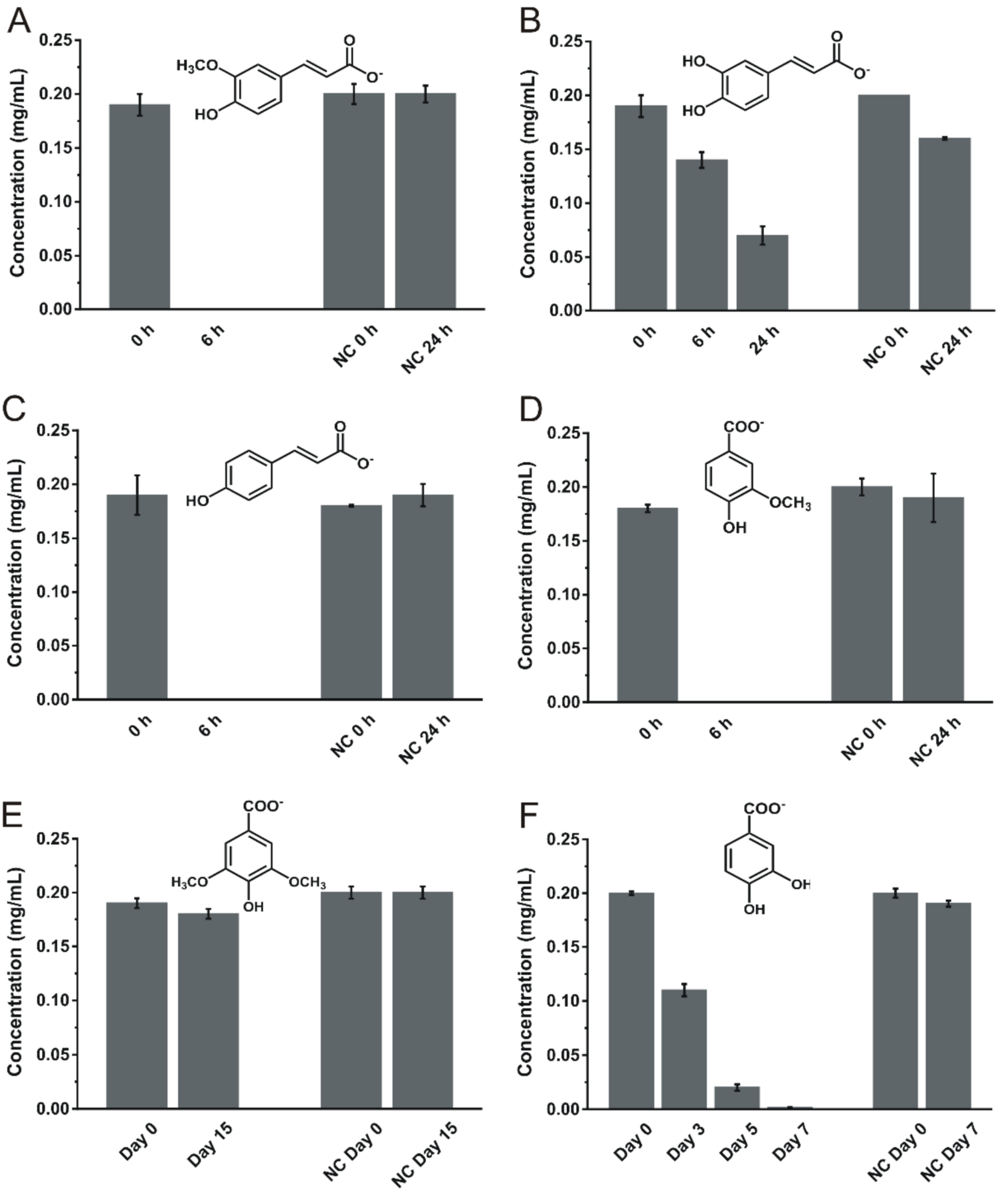

Figure 2. Biotransformation of hydroxycinnamates and hydroxybenzoates by *B. licheniformis* JCM 2505 type strain. Substrates used were ferulare (A), caffeate (B), *p*-coumarate (C), vanillate (D), syrigate (E) and protocatechuate (F). The bio-transformation pattern is the same with that shown by TAB7 indicating that TAB7 is not unique in its ability to metabolize tested phenolic compounds. Data are expressed as means ± standard deviation from triplicates. NC: Negative control.

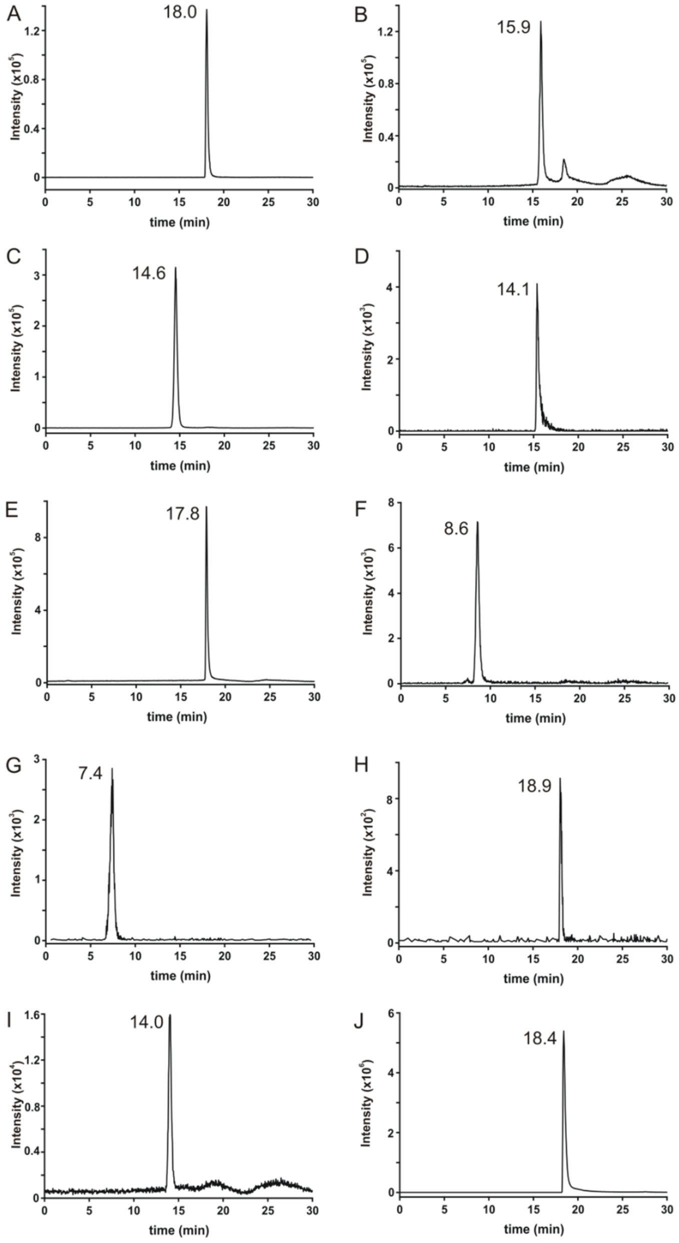

Figure 3. Ferulate (A), vanillin (B), caffeate (C), guaiacol (D), *p*-coumarate (E), catechol (F), protocatechuate (G), 4-vinylguaiacol (H), vanillate (I) and 4-ethylcatechol (J) standards run in LC-MS/MS using the method created for analysis of ferulate and caffeate bio-transformation intermediate/products by strain TAB7.

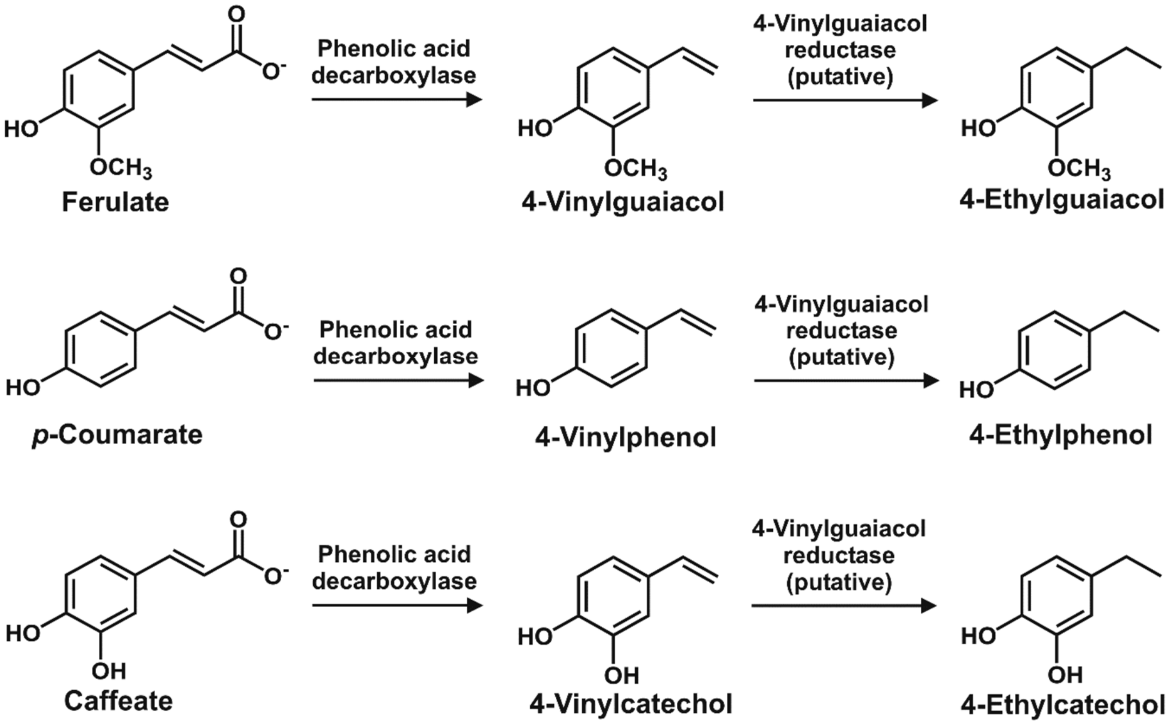

Figure 4. Conversion of ferulate, *p*-coumarate and caffeate into their vinyl and ethyl-derivatives. TAB7 could transfom ferulate, caffeate and *p*-coumarate and its genome harbors a putative phenolic acid decarboxylase. However, 4-vinylguaiacol could not be converted to its ethyl derivative while in caffeate biotransformation assay, 4-ethylcatechol was detected. This suggests that either the putative enzyme responsible for converting vinly derivatives into their corresponding ethyl forms in TAB7 has a stricter specificity or that it is induced by caffeate or one of its products.

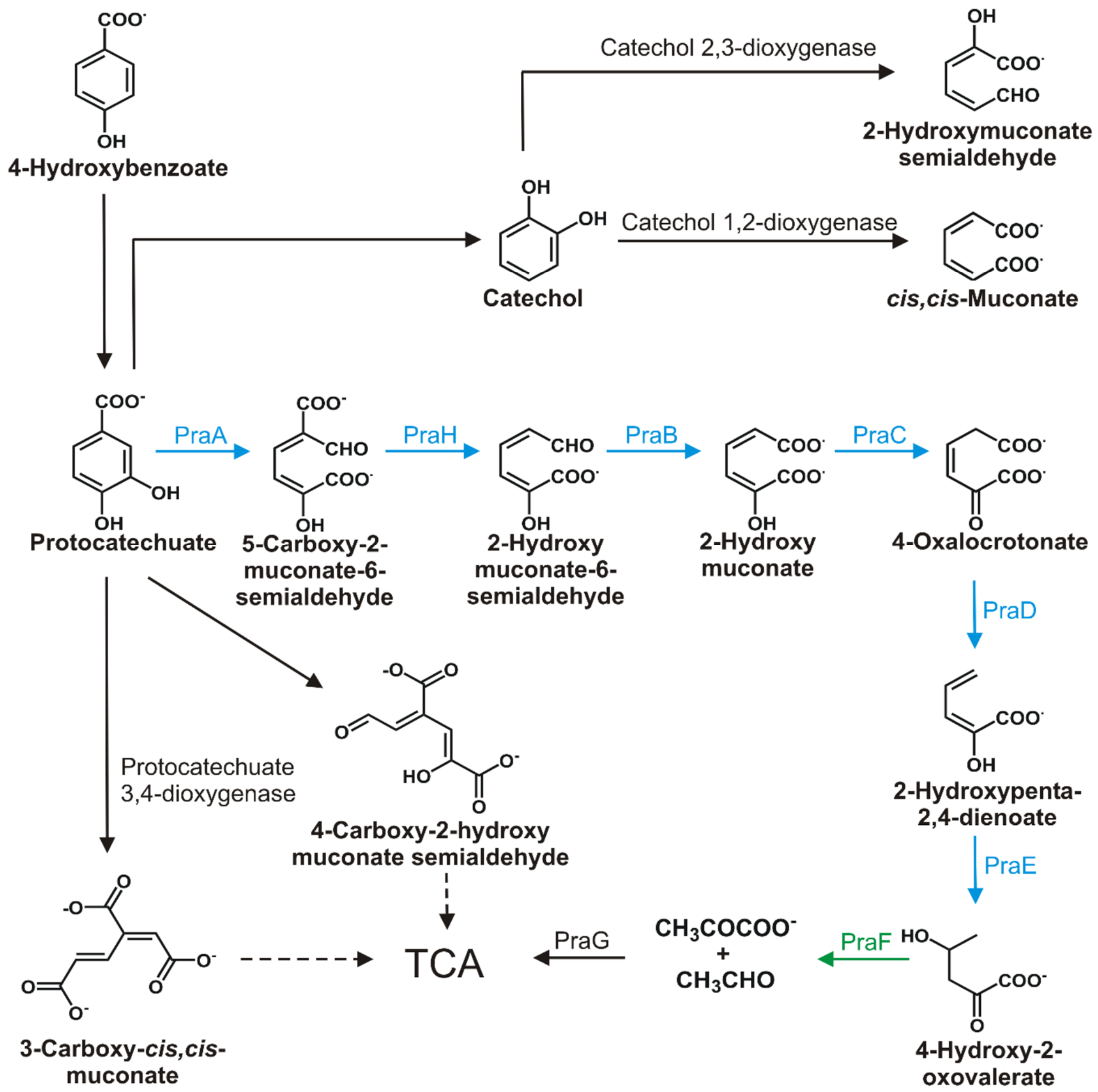

Figure 5. Protocatechuate degradation pathway found in TAB7. Blue arrows indicate the corresponding genes that were found in TAB7, while black arrows indicate genes not found in TAB7, and green arrow indicates the presence of a putative corresponding gene in TAB7 genome. Broken arrows indicate presence of more than one step.

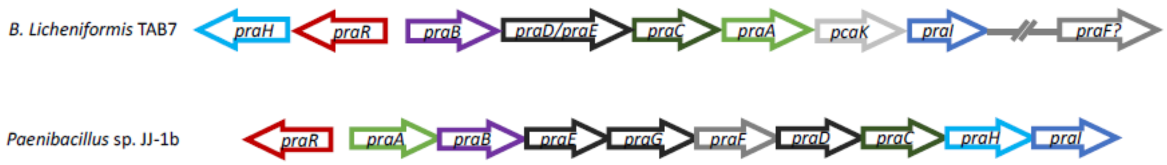

Figure 6. Gene organization of the *pra* gene cluster in *B. licheniformis* TAB7 and *Paenibacillus* sp. JJ- 1b. ORF are not drawn to scale. Gene designations are given in Table S1. Homologous genes are shown in identical colors. Putative PraF in TAB7 showed low homology (24% identity) with the query and was found further from the operon.

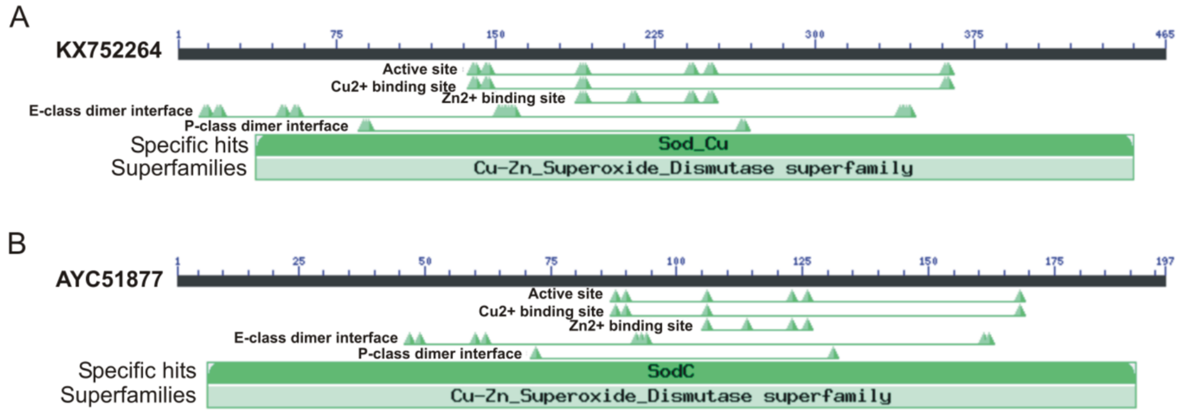

Figure 7. Comparison of conserved domains present in vinylphenol reductase/superoxide dismutase from *Brettanomyces bruxellensis* (GenBank: KX752264) (A) and putative vinylcatechol reductase/superoxide dismutase from strain TAB7 chromosome (Locus tag AYC51877) (B).

Table S1. Putative genes involved in degradation of protocatechuate by strain TAB7.

Protein / Enzyme	Gene	GenBank identifier	Organism	% Query cover	% ID	% Similarity	Locus Tag (TAB7)
Regulator	*praR*	BAH79098	*Paenibacillus* sp. JJ-1b	99	49	70	C7M53_21040
4-Hydroxybenzoate 3-hydroxylase	*praI*	BAH79107	*Paenibacillus* sp. JJ-1b	100	67	84	C7M53_21010
Protocatechuate 2,3-dioxygenase	*praA*	BAH79099	*Paenibacillus* sp. JJ-1b	99	57	76	C7M53_21020
5-Carboxy-2-hydroxymuconate-6-semialdehyde decarboxylase	*praH*	BAH79106	*Paenibacillus* sp. JJ-1b	99	59	75	C7M53_21045
2-Hydroxymuconate-6-semialdehyde dehydrogenase	*praB*	BAH79100	*Paenibacillus* sp. JJ-1b	98	75	88	C7M53_21035
4-Oxalocrotonate tautomarase	*praC*	BAH79105	*Paenibacillus* sp. JJ-1b	98	65	85	C7M53_21025
4-Oxalocrotonate decarboxylase	*praD*	BAH79104	*Paenibacillus* sp. JJ-1b	99	65	80	C7M53_21030
2-Hydroxypenta-2,4-dienoate hydratase	*praE*	BAH79101	*Paenibacillus* sp. JJ-1b	94	44	61	C7M53_21030
4-Hydroxy-2-oxovalerate aldolase	*praF*	BAH79103	*Paenibacillus* sp. JJ-1b	78	24	42	C7M53_10825
Acetaldehyde dehydrogenase	*praG*	BAH79102	*Paenibacillus* sp. JJ-1b		--	--	--
Protocatechuate decarboxylase	*aroY*	BAH20873	*Klebsiella pneumoniae* subsp. *pneumoniae*	93	29	43	C7M53_02095

Protocatechuat e 3,4-dioxygenase	*pcaGH*	AAD05270 AAD05269	*Streptomyces* sp. 2065	--	--	--
Protocatechuat e 4,5-dioxygenase	*ligAB*	BAB88742 BAB88743	*Sphingomonas paucimobilis*	--	--	--
